# Dialysis-Requiring Acute Kidney Injury Secondary to Severe Rhabdomyolysis in a Recreational Ketamine User

**DOI:** 10.7759/cureus.111521

**Published:** 2026-06-25

**Authors:** Jon Owen, Noel Fernando

**Affiliations:** 1 Acute Medicine, Princess Alexandra Hospital, Harlow, GBR; 2 Intensive Care Medicine, Princess Alexandra Hospital, Harlow, GBR

**Keywords:** drug and substance abuse, intensive care unit stay, ketamine-induced uropathy, rhabdomyolysis causing acute kidney injury, unplanned dialysis

## Abstract

Ketamine misuse is increasingly implicated in significant urological morbidity, most notably ulcerative cystitis and progressive bladder fibrosis. Rhabdomyolysis represents a less frequently recognised complication but carries the potential for life-threatening electrolyte derangement and acute kidney injury (AKI). We report a man in his mid-20s with heavy recreational ketamine use presenting after collapse with severe hyperkalaemia (8.2 mmol/L), marked rhabdomyolysis (peak creatine kinase ~40,000 IU/L), and stage 3 AKI requiring continuous venovenous haemodiafiltration (CVVHDF). Clinical management was further complicated by ketamine-associated bladder dysfunction, which precluded urethral catheterisation. This case study gives an overview of a patient who presented with a number of unusual features: severe ketamine-associated rhabdomyolysis, dialysis-requiring AKI, and complex electrolyte management on a background of established uropathy. We propose that renal failure was principally attributable to myoglobin-mediated acute tubular injury arising from rhabdomyolysis, potentiated by dehydration, prolonged immobilisation, sympathetic overactivity, and impaired urinary clearance.

## Introduction

Ketamine is a phencyclidine derivative introduced in 1964 as a dissociative anaesthetic. It acts predominantly as an N-methyl-D-aspartate (NMDA) receptor antagonist, with additional anticholinergic and GABA-modulating properties, producing analgesia, amnesia, and dissociation whilst generally preserving airway reflexes and haemodynamic stability [1].

Recreational use has risen substantially among young adults in the UK, with an estimated 252% rise in consumption between 2014-15 and 2024-25 [2]. Chronic misuse is strongly associated with ketamine-induced cystitis, characterised by urinary frequency, urgency, dysuria, haematuria, reduced bladder compliance, and progressive fibrosis [3-5]. Upper tract damage and renal impairment have been described in severe cases.

Rhabdomyolysis, by contrast, is infrequently reported in the context of ketamine toxicity, despite recreational drugs constituting one of the most common precipitant categories overall [6,7]. The condition arises from skeletal muscle necrosis via a broad range of mechanisms (psychomotor agitation, hyperthermia, prolonged immobilisation, and direct myotoxicity) with subsequent release of intracellular constituents, including creatine kinase (CK), potassium, phosphate, and myoglobin. Myoglobin-induced tubular toxicity and intraluminal obstruction may precipitate acute kidney injury (AKI) in 10%-59% of affected individuals [6]. Data from the European Drug Emergencies Network registry indicate that severe rhabdomyolysis occurs in approximately 2.4% of acute recreational drug toxicity presentations, with the risk of AKI increasing proportionally with the degree of biochemical derangement [7].

Although ketamine is less frequently implicated than stimulant substances, several published case reports document a temporal association between ketamine or esketamine exposure and clinically significant CK elevation. Tsai and colleagues described a patient with multiple trauma and crush injuries complicated by extreme exertion and ketamine consumption with CK concentrations exceeding 300,000 IU/L who required multiple haemodialysis sessions but ultimately achieved full renal recovery [8]. Zeiss et al. subsequently reported two cases of rhabdomyolysis following therapeutic esketamine administration, with CK values ranging from 8,000 to 22,000 IU/L that resolved following drug cessation [9]. Earlier emergency department case series have additionally documented milder degrees of rhabdomyolysis in agitated, ketamine-intoxicated individuals [10].

To date, only isolated case reports have described ketamine or esketamine in temporal association with severe rhabdomyolysis [8-10], and the underlying mechanisms remain incompletely understood. Below, we present a novel presentation of rhabdomyolysis and AKI in a recreational ketamine user with a range of features: severe rhabdomyolysis, dialysis-requiring AKI, profound hyperkalaemia, complex electrolyte management, and established ketamine uropathy.

## Case presentation

History

A 25-year-old male patient with an established history of heavy recreational ketamine use was found collapsed at his home following several days of progressive malaise. A precise timeline could not be established; however, the patient reported sustaining a fall with head trauma and being subsequently unable to mobilise for a prolonged period. He was discovered by a family member, having been incontinent of urine and having vomited extensively. The period of immobility was estimated at between 24 and 48 hours.

The patient disclosed a three-year history of heavy recreational ketamine misuse, with the onset of ketamine cystitis symptoms approximately 12 months prior to this presentation. He described these symptoms as severely debilitating, with urinary frequency sufficient to substantially impair both occupational function and social engagement. He reported episodic high-dose use during periods of low mood; on the occasion preceding this admission, he said that he had consumed an estimated 7 g of illicitly obtained ketamine, 3.5 g in the morning and 3.5 g in the evening, three days before presentation (although this could not be confirmed as point-of-care toxicological testing did not screen for ketamine indigestion). Regular medications prescribed for the management of ketamine cystitis included amitriptyline 10 mg three times daily, mirabegron 50 mg once daily, and solifenacin 5 mg once in the morning.

Initial investigations

Upon his initial presentation to the emergency department, his blood pressure was found to be 70/40 mmHg and fluid responsive. He had cold peripheries with a delayed capillary refill time. Glasgow Coma Scale (GCS) was 15. He presented with severe dysuria, passing urine approximately every two minutes, subjectively rating pain upon urination as 10/10.

Neurological assessment identified reduced power and sensation in both lower limbs, present from the morning of admission. Given a known history of significant nitrous oxide misuse, subacute combined degeneration of the spinal cord was considered a differential diagnosis; however, power and sensation resolved fully within two hours of presentation. CT imaging of the head demonstrated no acute intracranial pathology.

**Figure 1:**
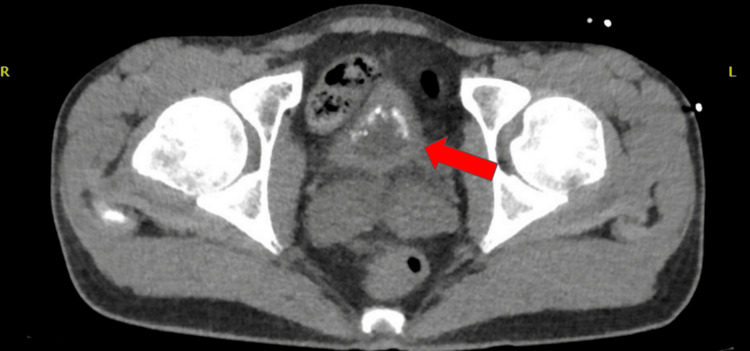
Axial CT (renal tract protocol) demonstrating bladder wall thickening with calcification (arrow).

**Figure 2:**
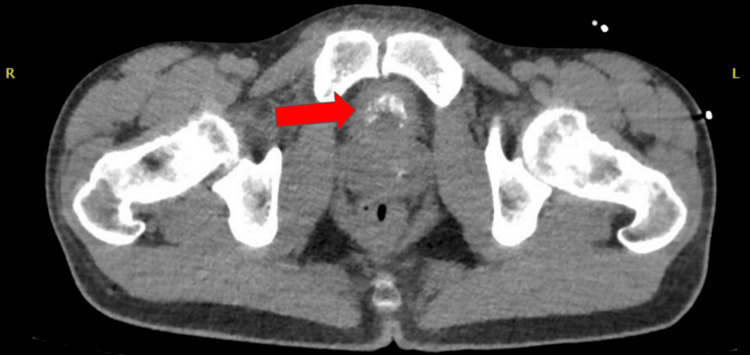
Axial CT (renal tract protocol) demonstrating prostatic calcification (arrow).

Multiple attempts at urethral catheterisation were undertaken but could not be completed due to intractable pain. CT imaging of the urinary tract demonstrated peripheral bladder wall calcification and significant prostatic calcification, findings considered likely to account for the failed catheterisation attempts. Bilateral small non-obstructing renal calculi (5 mm, right lower pole and left mid pole) were additionally identified (Figures 1, 2).

Biochemical investigations are demonstrated in Table 1.

**Table 1:** Biochemical investigations. eGFR: estimated glomerular filtration rate

	On admission	Admission + 12 hours	Normal range
Haemoglobin	173 g/L	157 g/L	130-170 g/L
White blood cells	25.3 x 10^9^/L	22.8 x 10^9^/L	3.0-10.0 x 10^9^/L
Platelet count	608 x 10^9^/L	746 x 10^9^/L	150-400 x 10^9^/L
Neutrophils	19.6 x 10^9^/L	21.5 x 10^9^/L	2.0-7.5 x 10^9^/L
Lymphocytes	1.2 x 10^9^/L	0.71 x 10^9^/L	1.5-4.0 x 10^9^/L
Monocytes	2.1 x 10^9^/L	3.1 x 10^9^/L	0.2-1.0 x 10^9^/L
Eosinophils	0.0 x 10^9^/L	0.0 x 10^9^/L	0-0.4 x 10^9^/L
Sodium	109 mmol/L	110 mmol/L	13 3-146 mmol/L
Potassium	8.6 mmol/L	7.6 mmol/L	3.4-5.3 mmol/L
Creatinine	387 µmol/L	348 µmol/L	60-120 µmol/L
eGFR	17 mL/min/1.73 m^2^	19 mL/min/1.73 m^2^	>60 mL/min/1.73 m^2^
Total bilirubin	28 µmol/L	35 µmol/L	<17 µmol/L
Alanine transaminase (ALT)	277 IU/L	292 IU/L	10-50 IU/L
Alkaline phosphatase (ALP)	959 IU/L	796 IU/L	25-115 IU/L
Albumin	45 g/L	40 g/L	35-50 g/L
Calcium	2.74 mmol/L	2.89 mmol/L	2.2-2.6 mmol/L
Phosphate	3.00 mmol/L	2.05 mmol/L	0.8-1.5 mmol/L
Magnesium	1.33	Not measured	0.7-1.0 mmol/L
C-reactive protein (CRP)	45 mg/L	40 mg/L	<5 mg/L
Creatinine kinase	28,155 IU/L	38.909 IU/L	25-200 IU/L

The overall clinical and biochemical picture was consistent with a diagnosis of severe rhabdomyolysis with secondary AKI, with haematuria attributable at least in part to traumatic catheterisation attempts.

Management

Hyperkalaemia was managed according to standard protocol with intravenous calcium chloride, insulin-dextrose infusion, nebulised salbutamol, potassium-binding therapy, and aggressive intravenous fluid resuscitation. Despite initial biochemical improvement, the combination of severe rhabdomyolysis, persistent hyperkalaemia, oliguric AKI, and extensive urinary tract calcification prompted transfer to the intensive care unit for renal replacement therapy.

Management was complicated by profound hyponatraemia (serum sodium 109 mmol/L) occurring concurrently with severe hyperkalaemia and dialysis-requiring AKI. Continuous venovenous haemodiafiltration (CVVHDF) was therefore commenced on day 2 of admission using a low-flow strategy to minimise the rate of sodium correction. Treatment was delivered using potassium-free replacement and dialysate solutions, with multiple 5 L exchanges administered over approximately 36 hours. Regional citrate anticoagulation was employed throughout therapy. To further limit sodium overcorrection, a concurrent 5% dextrose infusion was initiated at 100 mL/hour and subsequently titrated according to hourly serum sodium measurements. The target sodium concentration was maintained at no greater than 120 mmol/L during the first 24 hours. This approach enabled rapid correction of life-threatening hyperkalaemia whilst avoiding excessive sodium correction and the associated risk of osmotic demyelination syndrome.

**Figure 3:**
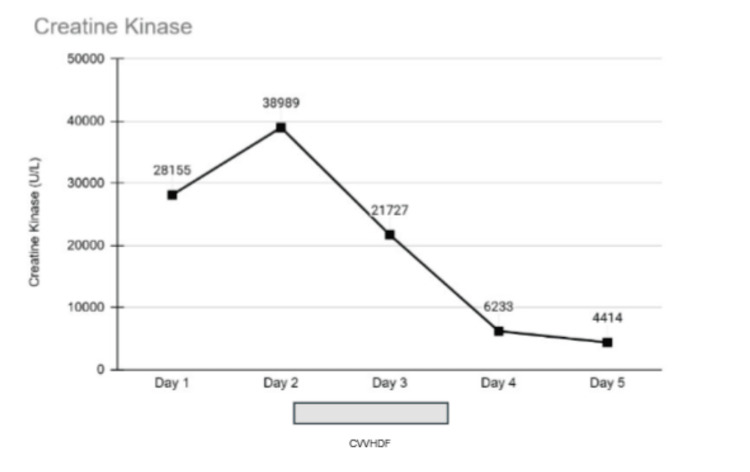
Creatinine kinase trend. CVVHDF: continuous venovenous haemodiafiltration

**Figure 4:**
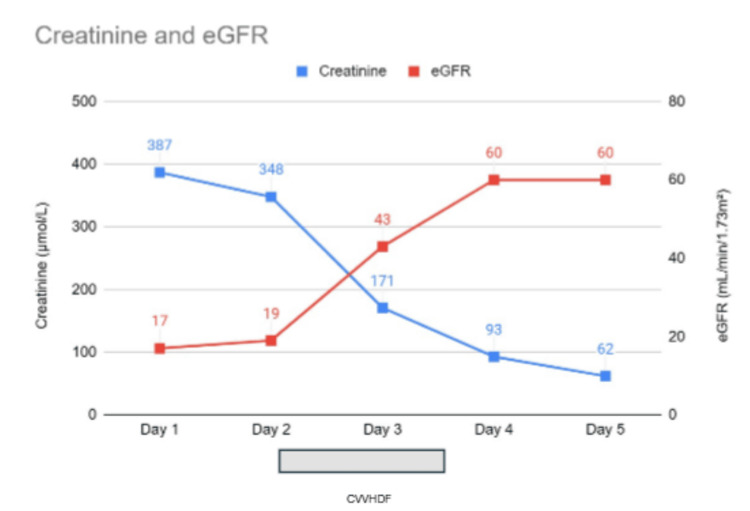
Creatinine and eGFR trend. eGFR values reported as >60 mL/min/1.73 m² by the laboratory were plotted as 60 mL/min/1.73 m² CVVHDF: continuous venovenous haemodiafiltration; eGFR: estimated glomerular filtration rate

CVVHDF continued from approximately 05:00 on day 2 until the afternoon of day 3, during which time serum potassium normalised and renal function improved substantially. Over the subsequent 72 hours, CK concentrations declined progressively from a peak of 38,989 U/L to 4,414 U/L, creatinine fell from 387 μmol/L to 62 μmol/L, and eGFR recovered from 17 mL/min/1.73 m² to greater than 60 mL/min/1.73 m². Renal replacement therapy was successfully discontinued, and the patient was stepped down from intensive care with arrangements for outpatient urological follow-up (Figures 3, 4).

## Discussion

The present case is distinguished by the severity of biochemical disturbance, including life-threatening hyperkalaemia, profound hyponatraemia, dialysis-requiring AKI, and the coexistence of established ketamine-associated lower urinary tract disease. The combination of severe rhabdomyolysis requiring continuous renal replacement therapy together with advanced ketamine uropathy appears to be rarely reported in the literature.

Complex electrolyte management

A notable feature of this case was the coexistence of severe hyperkalaemia (8.6 mmol/L) and profound hyponatraemia (109 mmol/L) in the setting of dialysis-requiring AKI. These abnormalities created competing therapeutic priorities. Whilst urgent renal replacement therapy was required to manage life-threatening hyperkalaemia, conventional dialysis risks excessively rapid correction of serum sodium, potentially precipitating osmotic demyelination syndrome.

The patient's profound hyponatraemia (serum sodium 109 mmol/L) was likely multifactorial in origin. Significant volume depletion resulting from vomiting, poor oral intake, prolonged immobilisation, and hypotension on presentation may have contributed through non-osmotic antidiuretic hormone (ADH) release. Although hyponatraemia is not a recognised complication of recreational ketamine misuse [1], concurrent exposure to other recreational substances, including MDMA (3,4-methylenedioxymethamphetamine), could not be excluded, as toxicological screening did not routinely test for these agents. Syndrome of inappropriate ADH secretion (SIADH) was not formally investigated, and therefore, a definitive mechanism could not be established.

To address this challenge of coexistent electrolyte disturbance, CVVHDF was delivered using a low-flow strategy with a potassium-free dialysate, whilst a concurrent 5% dextrose infusion was titrated against frequent biochemical monitoring to limit the rate of sodium correction. This facilitated effective potassium clearance whilst maintaining controlled sodium correction within accepted safety parameters. Although customised approaches to renal replacement therapy in severe hyponatraemia have been described, this case highlights the practical challenges encountered when life-threatening hyperkalaemia and profound hyponatraemia coexist.

Cause of AKI: rhabdomyolysis versus direct ketamine toxicity

Ketamine and its principal metabolites undergo renal elimination, and prolonged exposure has been associated with inflammatory and fibrotic injury of the lower urinary tract, with progression in some cases to obstructive uropathy and renal impairment [4,5]. Direct nephrotoxicity has consequently been proposed as a mechanism of kidney injury in heavy users. However, the clinical course in the present case is more consistent with rhabdomyolysis-induced acute tubular injury than with primary ketamine-mediated renal injury.

The patient's markedly elevated CK concentration, profound hyperkalaemia with electrocardiographic changes, and subsequent recovery following renal replacement therapy are characteristic of myoglobin-mediated AKI. In rhabdomyolysis, skeletal muscle necrosis releases myoglobin and intracellular electrolytes into the circulation; the haem moiety of myoglobin exerts direct tubular cytotoxicity and contributes to intrarenal vasoconstriction and tubular cast formation, culminating in acute tubular necrosis [6]. Epidemiological studies suggest that AKI complicates 10%-59% of rhabdomyolysis cases and accounts for a substantial proportion of severe presentations [6].

Importantly, although this patient had established ketamine-associated lower urinary tract disease, there was no radiological evidence of hydronephrosis or obstructive uropathy. Consequently, rhabdomyolysis remains the most likely primary mechanism underlying the AKI observed.

Mechanistic considerations

The pathogenesis of rhabdomyolysis in this patient was almost certainly multifactorial. Prolonged immobilisation following collapse likely resulted in sustained muscular compression and regional ischaemia, precipitating ATP depletion and pathological intracellular calcium accumulation. This final common pathway, characterised by failure of energy-dependent ion homeostasis with consequent calcium-mediated protease activation and mitochondrial dysfunction, is well established in the pathophysiology of rhabdomyolysis [6].

Ketamine intoxication itself may also have contributed through sympathetic nervous system activation and behavioural agitation. As an NMDA receptor antagonist, ketamine produces dissociative and hyperadrenergic effects that increase metabolic demand and skeletal muscle activity [1]. Psychomotor agitation and excessive muscular exertion are recognised precipitants of myonecrosis in toxicological presentations [7,10], and these effects may be amplified by concurrent polydrug use.

Concurrent dehydration and hypotension on admission likely further amplified the renal insult. Intravascular volume depletion reduces renal perfusion and impairs renal resilience to myoglobin-mediated injury, thereby increasing the likelihood of AKI [6,7].

The significance of the patient's coexisting ketamine-associated uropathy lies less in its direct contribution to renal failure and more in its demonstration of severe chronic ketamine toxicity. Extensive bladder and prostatic calcification complicated acute management by rendering urethral catheterisation extremely painful and ultimately unsuccessful. These findings highlight the substantially lower urinary tract morbidity that can accompany long-term ketamine misuse and may coexist with acute toxicological complications.

Collectively, the available evidence suggests that rhabdomyolysis constituted the primary renal insult in this case, with prolonged immobilisation, dehydration, hypotension, and ketamine intoxication acting as contributing factors. The concomitant presence of advanced ketamine-associated uropathy serves to illustrate the potential for acute and chronic complications of ketamine misuse to present simultaneously in a single patient.

## Conclusions

This case underscores that serum CK should be checked early in any intoxicated or immobilised patient presenting with AKI or hyperkalaemia, since electrolyte disturbance may precede overt recognition of rhabdomyolysis, and that early critical care involvement with timely renal replacement therapy can be life-saving in this setting. It also illustrates a specific technical challenge: when life-threatening hyperkalaemia coexists with profound hyponatraemia, continuous renal replacement therapy can be adapted using a low-flow, potassium-free strategy with a titrated dextrose infusion to achieve rapid potassium clearance whilst avoiding the risk of osmotic demyelination from overly rapid sodium correction.

Finally, this patient's presentation demonstrates that acute and chronic complications of ketamine misuse can coexist and compound one another clinically: severe rhabdomyolysis precipitated dialysis-requiring AKI, whilst established ketamine-associated uropathy independently caused painful urinary symptoms and rendered urethral catheterisation impossible. Clinicians managing ketamine-related presentations should therefore maintain a high index of suspicion for rhabdomyolysis even in patients with well-recognised chronic ketamine uropathy, as the two processes are mechanistically distinct but can present simultaneously.
